# The Ohsaki Cohort 2006 Study: Design of Study and Profile of Participants at Baseline

**DOI:** 10.2188/jea.JE20090093

**Published:** 2010-05-05

**Authors:** Shinichi Kuriyama, Naoki Nakaya, Kaori Ohmori-Matsuda, Taichi Shimazu, Nobutaka Kikuchi, Masako Kakizaki, Toshimasa Sone, Fumi Sato, Masato Nagai, Yumi Sugawara, Yasutake Tomata, Munira Akhter, Mizuka Higashiguchi, Naru Fukuchi, Hideko Takahashi, Atsushi Hozawa, Ichiro Tsuji

**Affiliations:** 1 Division of Epidemiology, Department of Public Health and Forensic Medicine, Tohoku University Graduate School of Medicine, Sendai, Japan; 2 Epidemiology and Prevention Division, Research Center for Cancer Prevention and Screening, National Cancer Center, Tokyo, Japan

**Keywords:** long-term care insurance, population-based, psychosocial factors, study design, the Ohsaki Cohort 2006 Study

## Abstract

**Background:**

Large-scale cohort studies conducted in Japan do not always include psychosocial factors as exposures. In addition, such studies sometimes fail to satisfactorily evaluate disability status as an outcome.

**Methods:**

This prospective cohort study comprised 49 603 (22 438 men and 27 165 women) community-dwelling adults aged 40 years or older who were included in the Residential Registry for Ohsaki City, Miyagi Prefecture, in northeastern Japan. The baseline survey, which included psychosocial factors, was conducted in December 2006. Follow-up of death, immigration, cause of death, cancer incidence, and long-term care insurance certification was started on 1 January 2007.

**Results:**

The response rate was 64.2%. In general, lifestyle-related conditions in the study population were similar to those of the general Japanese population; however, the proportion of male current smokers was higher in the cohort. The association between age and the proportion of those reporting psychological distress showed a clear U-shaped curve, with a nadir at age 60 to 69 years in both men and women, although more women were affected by such distress than men. The proportion of those who reported a lack of social support was highest among those aged 40 to 49 years. Most men and women surveyed did not participate in community activities. Among participants aged 65 years or older, 10.9% of participants were certified beneficiaries of the long-term care insurance system at baseline.

**Conclusions:**

The Ohsaki Cohort 2006 Study is a novel population-based prospective cohort study that focuses on psychosocial factors and long-term care insurance certification.

## INTRODUCTION

Increasing evidence suggests that, in addition to biomedical factors, a broad range of psychosocial factors influences general health.^1^^–^^3^ However, large-scale cohort studies performed in Japan may not have sufficiently considered these factors as exposures in evaluating health outcomes.^4^^–^^9^

In addition to this tendency to overlook psychosocial exposures, some types of health outcomes, such as disability status, have not been satisfactorily examined in large-scale epidemiological studies in Japan.^4^^–^^9^ Although there is growing concern about the quality of life of seniors,^10^^,^^11^ assessment of quality of life—in particular disability status—by means of general population surveys presents many challenges.^12^^–^^14^ In 2000, the Japanese government implemented a mandatory social long-term care insurance (LTCI) system to promote the independence of seniors by facilitating access to appropriate high-quality services of their choice, whenever and wherever needed.^15^^,^^16^ Therefore, there is now an opportunity to use LTCI certification status as an alternative to the evaluation of physical and mental disability.

Based on the need for a novel cohort that accounts for the recent diversification in the abovementioned exposures and outcomes, we initiated a large population-based prospective cohort study, the Ohsaki Cohort 2006 Study, the main objective of which is to examine the association between psychosocial factors and both physical and mental disability status. Here, we report the design of the study and the profile of participants at baseline.

## METHODS

### Study design, setting, and participants

In this prospective cohort study, the source population for the baseline survey comprised community-dwelling individuals aged 40 years or older who were included in the Residential Registry for Ohsaki City, Miyagi Prefecture, northeastern Japan, as of 1 December 2006. The Residential Registry identified 78 101 persons (36 397 men; 41 704 women) in the area.

The baseline survey was conducted from 1 December to 15 December 2006. A questionnaire was distributed by the heads of individual administrative districts to individual households, after which it was collected by mail.

### Baseline survey

The baseline questionnaire for persons aged 40 to 64 years requested information on the following, in sequence: (1) history of diseases, (2) family history of diseases, (3) health status over the last year, (4) smoking status, (5) alcohol drinking status, (6) dietary habits,^17^ (7) job status and educational status, (8) present and past body weight and height, (9) general health status, (10) sports and exercise,^18^^,^^19^ (11) psychological distress (using the K6, a 6-item instrument that assesses nonspecific psychological distress developed by Kessler and colleagues),^20^^–^^23^ (12) social support,^24^ (13) participation in community activities, (14) dental status, and (15) reproductive factors (in women).

Question items for persons aged 65 years or older were the same as those for persons aged 40–64, excluding family history of diseases, job status and educational status, present and past body weight and height, and reproductive factors. In addition, we included a frailty checklist (the Kihon Checklist, in Japanese),^25^ along with (1) past body weight and height, (2) pain, and (3) daily activities. The Kihon Checklist is a tool developed by the Japanese Ministry of Health, Labour, and Welfare to screen for frailty, and is designed to measure actual task performance.^25^

All people who supplied their name and address, and completed most of the questionnaire, were regarded as eligible; all others were excluded. The reasonableness of data was evaluated according to predetermined rules.

### Follow-up

We conducted this prospective cohort study with the cooperation of the Ohsaki City municipal government after obtaining their written agreement. The aim is to follow the cohort participants for mortality and immigration using the Residential Registry of Ohsaki City. We also confirm information regarding LTCI certification status among individuals aged 65 years or older, after obtaining written consent for review of these data. Causes of death are confirmed by review of death certificates, with approval from the Japanese Ministry of Internal Affairs and Communications and the Japanese Ministry of Health, Labour, and Welfare. Cancer incidence is also confirmed by review of data from the Miyagi Prefectural Cancer Registry, with approval from the Miyagi Prefectural Cancer Registry Committee.

### Ethical issues

The return of questionnaires completed by the participants was regarded as consent to participate in the study, which involves cross-sectional analysis of baseline survey data and information on subsequent mortality and immigration. We provided an explanatory note on the questionnaire that stated we would follow the cohort participants for mortality and cancer incidence. The study protocol was reviewed and approved by the Ethics Committee of Tohoku University Graduate School of Medicine.

## RESULTS

Data on the source population, eligible population, study population, and response rate by age and sex are shown in Table 1. Of the 78 101 people in the source population, we were unable to contact 866, yielding an eligible population of 77 235. Baseline questionnaires were collected from 50 210 persons, and valid responses were received from 49 603 (22 438 men and 27 165 women), who formed the study population of cohort participants. Among the invalid responses, 252 persons aged 65 years or older completed the questionnaires intended for those aged 40 to 64 years. Among the study population, 26 512 persons (53.4%) were aged 40 to 64 years, and 23 091 (46.6%) were aged 65 years or older. The response rate was calculated by dividing the study population by the total eligible population, yielding 64.2%. The response rate for men was 62.4% (22 438/35 965), and was somewhat lower than that for women, at 65.8% (27 165/41 270). By age, the response rate for persons aged 65 years or older was high, at 73.9% (23 091/31 237), while that for persons aged 40 to 64 years was 57.6% (26 512/45 998).

**Table 1.** Response rate and number of adults in source population, eligible population, and study population

	Aged 40–64 years		Aged ≥65 years		Total
		
	Men	Women	Men	Women	
No. of source population	23 647	22 760	12 750	18 944	78 101
No. of eligible population (A)	23 359	22 639	12 606	18 631	77 235
No. of persons responding to the survey	12 967	13 849	9690	13 704	50 210
No. of study population (B)	12 833	13 679	9605	13 486	49 603
Response rate (B/A) (%)	54.9	60.4	76.2	72.4	64.2

### Selected baseline medical and lifestyle-related profiles of the study population

The selected baseline medical and lifestyle-related profiles of the study population are shown in Table 2. The prevalence of a history of serious disease rose with increasing age in both men and women. In men, the distributions of a history of hypertension, diabetes mellitus, and cancer all peaked at age 70 to 79 years. More than 40% of men aged 75 to 79 years had a history of hypertension. About 60% of men, and 20% of women, aged 40 to 44 years currently smoked, and more than 80% of men, and 50% of women, in the same age group currently drank alcohol at baseline, which decreased with increasing age. The proportion of obese individuals, defined as a BMI ≥25.0 kg/m^2^, was inversely associated with age in men, but weakly positively associated with age in women, with a peak at age 70 to 79 years. The association between age and the proportion of individuals who were underweight, defined as a BMI <18.5 kg/m^2^, was J-shaped for men and U-shaped for women. The association between age and the proportion of those who spent less than 1 hour per day walking was J-shaped for both men and women.

**Table 2.** Selected baseline medical and lifestyle-related profiles of study population, by sex and age category

Variables	Age category (years)									

	40–44	45–49	50–54	55–59	60–64	65–69	70–74	75–79	80–84	≥85
**Men**										
No. of participants	1857	2365	2884	3427	2300	2477	2846	2391	1256	635
History of serious disease (%)										
Hypertension	7.7	14.6	18.8	25.4	32.7	37.8	41.9	44.0	39.7	35.3
Diabetes mellitus	3.6	5.2	7.5	11.0	12.8	14.4	16.2	13.6	11.5	10.1
Stroke	0.4	0.6	1.3	2.0	3.4	4.4	5.6	7.1	7.9	8.8
Myocardial infarction	0.1	0.7	1.0	1.8	3.5	4.7	5.9	8.5	9.7	11.5
Cancer	0.8	1.5	2.1	2.8	5.0	7.4	10.4	13.0	12.3	9.8
Current smokers (%)	59.5	56.7	50.6	46.8	40.4	31.4	25.9	21.3	19.2	11.1
Current alcohol drinkers (%)	81.5	80.6	80.7	79.2	77.0	69.1	61.6	53.2	45.3	30.3
Body mass index (%)										
<18.5 kg/m^2^	2.7	2.5	1.7	2.0	2.5	3.0	3.7	6.2	11.0	10.9
≥25.0 kg/m^2^	35.1	33.8	34.7	34.7	30.8	32.1	29.1	26.3	19.7	16.6
Time spent walking <1 hr/day (%)	69.4	68.4	67.5	67.2	67.3	63.9	67.9	74.2	79.0	85.3

**Women**										
No. of participants	1935	2488	3025	3638	2593	3070	3623	3303	2021	1469
History of serious disease (%)										
Hypertension	3.3	8.3	15.0	23.5	30.1	37.0	43.0	46.4	47.7	46.1
Diabetes mellitus	0.8	2.6	3.3	6.0	8.4	8.5	10.4	11.6	12.0	10.2
Stroke	0.3	0.2	0.6	0.5	1.1	1.6	2.5	3.8	4.6	6.3
Myocardial infarction	0.1	0.0	0.2	0.5	1.0	1.5	2.9	4.4	6.1	7.2
Cancer	2.0	2.7	4.3	4.7	6.9	6.0	5.9	6.2	6.9	7.9
Current smokers (%)	19.6	15.2	11.0	9.1	7.3	4.7	3.8	2.8	2.5	2.1
Current alcohol drinkers (%)	56.7	49.5	40.3	34.1	29.9	20.7	14.4	11.6	10.8	9.2
Body mass index (%)										
<18.5 kg/m^2^	7.5	6.3	4.8	4.2	3.3	3.7	4.8	6.4	9.1	16.1
≥25.0 kg/m^2^	20.1	22.6	27.4	28.3	32.2	34.9	35.2	31.9	27.7	22.0
Time spent walking <1 hr/day (%)	74.0	70.3	70.2	71.6	73.4	70.0	72.5	78.8	84.4	91.6

### Selected baseline psychosocial profiles of the study population

With regard to psychosocial profiles (Table 3), the association between age and the proportion of participants who had psychological distress showed a clear U-shaped curve in both sexes, with a nadir in those aged 60 to 69 years; psychological distress was more common in women than in men. The proportion of those who reported lack of social support was highest among those in their 40s, and decreased with age for every component of social support in both men and women. More men than women reported lack of social support. About 20% of men in their 40s reported lack of social support for consultation when in trouble. In contrast, the association between age and the proportion of those who did not participate in community activities showed a J-shape curve with a nadir at age 60 to 69 years.

**Table 3.** Selected baseline psychosocial profiles of study population, by sex and age category

Variables	Age category (years)									

	40–44	45–49	50–54	55–59	60–64	65–69	70–74	75–79	80–84	≥85
**Men**										
Psychological distress^a^, yes (%)	7.1	7.4	6.2	5.1	4.7	4.1	4.8	5.7	7.0	6.9
Lack of social support (%)										
(i) To consult when you are in trouble	19.0	20.1	18.0	18.0	18.1	14.5	12.7	13.3	13.1	13.7
(ii) To consult when you are in bad physical condition	15.8	15.7	15.1	13.9	12.6	8.9	8.0	7.7	5.9	7.4
(iii) To help with your daily housework	18.2	18.9	17.8	17.5	18.8	17.6	15.8	16.2	13.0	9.0
(iv) To take you to a hospital	13.5	12.3	11.7	9.5	9.5	7.8	7.6	8.0	6.9	5.3
(v) To take care of you	10.8	11.0	11.6	9.6	10.5	8.8	9.1	9.0	9.9	8.9
No participation in community activities (%)										
(i) Neighborhood association activities	50.1	46.0	45.8	44.6	45.5	42.9	48.0	50.2	59.5	70.6
(ii) Sports or exercise	47.9	49.6	51.6	53.9	49.5	45.8	50.3	55.4	61.7	70.9
(iii) Volunteering	69.3	63.6	61.4	60.4	60.5	56.1	60.8	65.1	75.6	88.7
(iv) Social gatherings	52.7	53.0	50.8	48.5	46.3	40.3	44.9	50.7	61.0	78.9

**Women**										
Psychological distress^a^, yes (%)	9.9	8.7	7.4	6.6	5.4	5.3	6.4	7.5	10.5	13.9
Lack of social support (%)										
(i) To consult when you are in trouble	11.1	10.8	10.2	10.5	9.9	7.9	7.4	7.1	7.7	4.9
(ii) To consult when you are in bad physical condition	11.5	10.8	9.2	9.1	8.9	6.3	5.8	5.1	4.8	2.7
(iii) To help with your daily housework	16.5	15.4	13.5	16.2	16.8	15.3	15.7	13.0	9.7	4.5
(iv) To take you to a hospital	13.3	11.6	7.5	8.8	8.2	8.0	7.8	7.4	5.5	3.4
(v) To take care of you	16.5	15.6	13.4	16.4	17.3	17.8	19.0	17.1	12.9	7.0
No participation in community activities (%)										
(i) Neighborhood association activities	42.3	52.0	57.3	56.3	54.2	53.7	55.8	59.5	69.8	86.8
(ii) Sports or exercise	61.1	58.8	60.5	56.4	51.4	50.0	54.1	62.5	75.0	88.3
(iii) Volunteering	78.8	75.7	73.7	70.1	67.3	67.0	74.3	81.0	88.9	97.4
(iv) Social gatherings	59.5	59.7	60.3	56.9	53.2	51.1	56.2	61.0	74.0	91.9

^a^The K6 was used as an indicator of psychological distress.^20^^–^^23^

### LTCI certification at baseline

The percentages of participants aged 65 years or older at baseline who received LTCI certification are shown in Table 4. Among participants in this age group, 16 739 (72.5%) provided written consent for our review of the information. Among these seniors, 10.9% had been LTCI-certified as of 15 December 2006. The proportion of those who were LTCI-certified increased linearly in relation to age category in both men and women; more women were LTCI-certified than men. Among participants aged 85 years or older, about 34% of men and 51% of women were LTCI-certified.

**Table 4.** Number (%) of participants certified in the long-term care insurance system of Japan at baseline

Care level	Age category (years)				

	65–69	70–74	75–79	80–84	≥85
**Men**					
Uncertified	1817 (97.6)	2037 (95.5)	1683 (92.6)	808 (85.0)	316 (65.8)
Support level 1^a^	4 (0.2)	7 (0.3)	13 (0.7)	14 (1.5)	12 (2.5)
Support level 2^a^	4 (0.2)	17 (0.8)	16 (0.9)	22 (2.3)	11 (2.3)
Care level 1^b^	10 (0.5)	21 (1.0)	27 (1.5)	23 (2.4)	47 (9.8)
Care level 2^b^	15 (0.8)	11 (0.5)	25 (1.4)	34 (3.6)	30 (6.3)
Care level 3^b^	3 (0.2)	15 (0.7)	20 (1.1)	28 (2.9)	27 (5.6)
Care level 4^b^	5 (0.3)	18 (0.8)	25 (1.4)	11 (1.2)	18 (3.8)
Care level 5^b^	4 (0.2)	6 (0.3)	9 (0.5)	11 (1.2)	19 (4.0)

**Women**					
Uncertified	2153 (98.3)	2411 (95.0)	2076 (90.2)	1090 (77.2)	520 (49.4)
Support level 1^a^	4 (0.2)	24 (0.9)	41 (1.8)	49 (3.5)	36 (3.4)
Support level 2^a^	7 (0.3)	31 (1.2)	45 (2.0)	52 (3.7)	59 (5.6)
Care level 1^b^	9 (0.4)	25 (1.0)	57 (2.5)	92 (6.5)	126 (12.0)
Care level 2^b^	3 (0.1)	13 (0.5)	26 (1.1)	48 (3.4)	93 (8.8)
Care level 3^b^	8 (0.4)	10 (0.4)	20 (0.9)	28 (2.0)	83 (7.9)
Care level 4^b^	5 (0.2)	15 (0.6)	22 (1.0)	29 (2.1)	70 (6.7)
Care level 5^b^	2 (0.1)	10 (0.4)	15 (0.7)	24 (1.7)	65 (6.2)

^a^Those who require support for daily activities; a higher number indicates a need for greater support.

^b^Those who require continuous care; a higher number indicates a need for greater continuous care.

## DISCUSSION

To characterize the study population, we compared selected health-related characteristics of the population with those of the Japanese general population, by sex and age, using data from The National Health and Nutrition Survey in Japan, 2005.^26^ Among men, the proportion of current smokers was higher in the study population than in the general population. The proportions of current smokers at baseline in the present cohort population by age category were 56.7% to 59.5%, 46.8% to 50.6%, 31.4% to 40.4%, and 21.3% to 25.9% for men in their 40s, 50s, 60s, and 70s, respectively (Table 2); the corresponding figures from the national survey were 44.1%, 42.5%, 34.0%, and 20.0% (≥70 years). In contrast, smoking status among women in the study population was very similar to that in the general population. Other variables, including obesity, underweight, history of serious diseases, alcohol drinking, and time spent walking, were similarly prevalent among middle-aged and elderly men and women in the study population and general population. To take one example, the proportions of men who were obese (BMI of ≥25.0) at baseline in the present cohort population by age category were 33.8% to 35.1%, 34.7%, 30.8% to 32.1%, and 26.3% to 29.1% for those in their 40s, 50s, 60s, and 70s, respectively (Table 2); the corresponding figures from the national survey were 34.1%, 31.4%, 30.7%, and 26.0% (≥70 years), respectively.

We also compared the LTCI certification status of the participants with that of the Japanese population by sex and age.^27^ The proportions of those certified at baseline in the present cohort population, by age category, were 2.4%, 4.5%, 7.4%, 15.0%, and 34.2% for men aged 65–69, 70–74, 75–79, 80–84, and ≥85 years (Table 4); the corresponding figures from the estimated national survey were 3.0%, 6.2%, 11.9%, 22.1%, and 45.0%, respectively.^27^ The same comparison among women yielded similar results, with smaller proportions in the present cohort population. These observed smaller proportions were not unexpected, because people with disabilities have more difficulties in responding to questionnaires. However, the small magnitude of the difference indicates that the selection bias was not serious.

Our study had some limitations. First, the response rate (64.2%) was not very high. The response rates of men and women aged 40 to 64 years were lower (54.9% and 60.4%, respectively) than those of men and women aged 65 years or older (76.2% and 72.4%, respectively). These relatively low response rates, especially among participants aged 40 to 64 years, should be kept in mind when interpreting the study results. Second, among the psychosocial variables studied, the items regarding job status and educational status, social support, and participation in community activities have not been adequately validated. Third, LTCI certification does not directly indicate an individual’s disability status; however, it does reflect the burden of disability on society.^15^^,^^16^

We have already conducted a prospective cohort study in the catchment area of Ohsaki Public Health Center. This study began in 1995 and was named the Ohsaki National Health Insurance (NHI) beneficiary’s Cohort Study, or the Ohsaki Cohort Study.^5^ The primary purpose of that study was to demonstrate quantitatively the economic impact of health-related lifestyles; the Ohsaki Cohort 2006 Study, in contrast, does not assess medical costs. The catchment area of the Ohsaki Public Health Center included Furukawa City, and the towns of Nakaniida, Onoda, Miyazaki, Shikama, Matsuyama, Sanbongi, Kashimadai, Iwadeyama, Naruko, Wakuya, Tajiri, Kogota, and Nango. Among these areas, the city of Furukawa, and the towns of Matsuyama, Sanbongi, Kashimadai, Iwadeyama, Naruko, and Tajiri were consolidated to form the city of Ohsaki on 31 March 2006. The population of the present study and that investigated in the Ohsaki Cohort overlap by about one-third.

In conclusion, we have begun a large population-based prospective study that focuses on psychosocial factors and LTCI certification status. The psychological factors include measurements of job status and educational status, psychological distress,^20^^–^^23^ social support,^24^ participation in community activities, and the Kihon Checklist.^25^ LCTI certification is followed up as an alternative to individual disability status, and as a measure of the economic burden of disability on society.
